# Pre-existing malignancy results in increased prevalence of distinct populations of CD4^+^ T cells during sepsis

**DOI:** 10.1371/journal.pone.0191065

**Published:** 2018-01-16

**Authors:** Jianfeng Xie, Jennifer M. Robertson, Ching-wen Chen, Wenxiao Zhang, Craig M. Coopersmith, Mandy L. Ford

**Affiliations:** Department of Surgery, Emory University, Atlanta, GA, United States of America; University of Kentucky, UNITED STATES

## Abstract

The presence of pre-existing malignancy in murine hosts results in increased immune dysregulation and risk of mortality following a septic insult. Based on the known systemic immunologic changes that occur in cancer hosts, we hypothesized that the presence of pre-existing malignancy would result in phenotypic and functional changes in CD4^+^ T cell responses following sepsis. In order to conduct a non-biased, unsupervised analysis of phenotypic differences between CD4^+^ T cell compartments, cohorts of mice were injected with LLC1 tumor cells and tumors were allowed to grow for 3 weeks. These cancer hosts and age-matched non-cancer controls were then subjected to CLP. Splenocytes were harvested at 24h post CLP and flow cytometry and SPADE (Spanning-tree Progression Analysis of Density-normalized Events) were used to analyze populations of CD4^+^ cells most different between the two groups. Results indicated that relative to non-cancer controls, cancer mice contained more resting memory CD4^+^ T cells, more activated CD4^+^ effectors, and fewer naïve CD4^+^ T cells during sepsis, suggesting that the CD4^+^ T cell compartment in cancer septic hosts is one of increased activation and differentiation. Moreover, cancer septic animals exhibited expansion of two distinct subsets of CD4^+^ T cells relative to previously healthy septic controls. Specifically, we identified increases in both a PD-1^hi^ population and a distinct 2B4^hi^ BTLA^hi^ LAG-3^hi^ population in cancer septic animals. By combining phenotypic analysis of exhaustion markers with functional analysis of cytokine production, we found that PD-1^+^ CD4^+^ cells in cancer hosts failed to make any cytokines following CLP, while the 2B4^+^ PD-1^lo^ cells in cancer mice secreted increased TNF during sepsis. In sum, the immunophenotypic landscape of cancer septic animals is characterized by both increased CD4^+^ T cell activation and exhaustion, findings that may underlie the observed increased mortality in mice with pre-existing malignancy following sepsis.

## Introduction

Sepsis is the leading cause of death among critically ill patients in the United States with between 270,000 and 380,000 people dying of the disease annually [1]. Patients with malignancy are nearly ten times more likely to develop sepsis than the general population [2], and cancer represents the most common co-morbidity in septic patients [3–5]. Sepsis is also the leading cause of ICU admission in patients with cancer [6, 7]. Importantly, cancer is also the co-morbidity associated with the highest risk of death in sepsis, and hospital mortality can exceed 50% in patients with cancer and sepsis or septic shock [5, 7–10]. The etiology behind the increased mortality seen in cancer patients who develop sepsis compared to healthy patients who develop sepsis is multifactorial [10, 11]. While some deaths are secondary to immunosuppression related to cancer treatment (chemotherapy, radiation), others are likely related to a reduced ability of the host to develop an adaptive response to infection in the setting of chronic systemic changes related to the underlying malignancy. The two types of solid tumors that are associated with the highest incidence of sepsis are pancreatic cancer, at a rate of over 14,000 cases per 100,000 patients, and lung cancer, which has a rate of over 4600 cases per 100,000 patients [10]. We have established and published on models using both of these tumor types in septic mice [12–14], and both revealed a ~ 3-fold increase in mortality in cancer sepsis as compared to sepsis alone, suggesting that these are clinically relevant models in which the increased risk of death is similar to that observed in cancer patients who develop sepsis. In our previous publication in which we first described the increased mortality in cancer septic animals as compared to sepsis alone, we made the observation that cancer septic mice had alterations in both the number and frequency of splenic CD4^+^ T cells along with altered CD4^+^ T cells apoptosis, but exhibited no changes in splenic CD8^+^ T cell numbers [14]. Moreover, cancer septic animals exhibited higher bacterial burden in the peritoneal cavity, but this was not associated with alterations in local or systemic cytokines, neutrophil or dendritic cell responses [13, 14]. Thus, in this manuscript we have endeavored to interrogate the phenotype and functionality of CD4^+^ T cell responses in cancer septic hosts.

Emerging evidence over the last decade strongly points to a role for T cell coinhibitory molecules in mediating immune dysregulation during sepsis. Coinhibitory molecules including PD-1 and BTLA have been identified on the surface of T cells isolated from septic patients as opposed to those obtained from non-septic controls [15], and blockade of these pathways may represent a therapeutic strategy for the amelioration of morbidity and mortality in septic individuals [16–24]. In particular, many published studies demonstrate that anti-PD-1 and anti-PD-L1 improve survival in murine models of sepsis [16, 20, 25–32], and a clinical trial of anti-PD-L1 in sepsis is nearing completion (clinicaltrials.gov #NCT02576457). In addition, we recently identified a cell-intrinsic role for 2B4 on CD4^+^ T cells in increasing immune dysfunction and mortality during sepsis [33].

While it is well known that lymphocytes in cancer microenvironments also tend to exhibit an exhausted phenotype [34–36], which manifests in part in the co-expression of several distinct coinhibitory receptors including PD-1, BTLA, and 2B4, the systemic immune changes that occur as a result of malignancy and their impact on immune responses during infection have not been well characterized. To further understand how pre-existing malignancy influences systemic immune responses, we recently conducted analysis of immune compartments in cancer vs. non-cancer hosts in the absence of sepsis using a murine lung cancer model (LLC1). Splenocytes were stained for coinhibitory receptors 2B4, BTLA, and PD-1 in LLC1 tumor-containing animals and unmanipulated controls. Compared to mice without cancer, mice with cancer possessed increased frequencies and absolute numbers of 2B4^+^, BTLA^+^, and PD-1^+^ T cells [37]. However, the fate of these cells following sepsis was unknown. Here we postulated that these differences in systemic immunity in cancer hosts are associated with the altered CD4^+^ T cell response during sepsis. To test this, we interrogated the differences in both immunologic phenotype and function of CD4^+^ T cell populations in cancer septic animals as compared to previously healthy septic hosts.

## Materials and methods

### Mice

Six-week-old male and female B6 mice were purchased from the Jackson Laboratory. All animals were maintained in accordance with Emory University Institutional Animal Care and Use Committee guidelines. This study was approved by the Emory University IACUC (Protocol: DAR-2003199-071418BN). Mice were randomized to cancer and non-cancer groups. Three weeks after mice receive tumor cells, both groups received cecal ligation and puncture (CLP). To minimize animal suffering, all mice received pre-operative analgesia with Buprenex (0.1 mg/kg, McKesson Medical, San Francisco, CA). Animals were sacrificed 24 hours after CLP by CO2 inhalation.

### Cancer model

Murine lung cancer was induced via a subcutaneous injection of a murine lung carcinoma cells (LLC1) in the right inner thigh and tumors were allowed to grow for three weeks. LLC1 was cultured in RPMI 1640 medium supplemented with 10% fetal bovine serum, 1% glutamine, and 1% penicillin-streptomycin and 1%4-(2-hydroxyethyl) -1-piperazineethanesulfonic acid (HEPES). Each mouse in the cancer group received 250,000 LLC1 cells suspended in 0.1mL of phosphate buffered saline. Beginning seven days post inoculation, mice were checked daily for tumor growth. Animals were sacrificed if the tumor size became larger than 2 centimeters. Three weeks after injection, mice were subjected to CLP. Age and gender-matched “previously healthy” control mice were unmanipulated prior to CLP.

### Cecal ligation and puncture

The CLP model of septic peritonitis was used both in cancer and previously healthy mice. Injury was titrated to achieve a ~50% 14-day mortality in previously healthy septic mice to mimic the clinical scenario of sepsis [38]. In brief, C57BL/6 mice were anesthetized using isoflurane and underwent laparotomy, the cecum was exteriorized, ligated distal to the ileocecal valve, and punctured twice with a 25-gauge needle. Sham-operated animals underwent laparotomy and exteriorization of the cecum only. Personnel conducting sham and CLP surgeries received training and competency testing from Emory University Division of Animal Resources veterinary staff. All animals received buprenorphine (0.1mg/kg) preoperatively for pain relief and 1mL of normal saline for intraoperative fluid losses as well as antibiotics (ceftriaxone 25mg/kg and metronidazole 12.5mg/kg) subcutaneously postoperatively. Antibiotics were continued on a q12hr dosing schedule for 48 hours postoperatively. Animals were observed every 12 hours following surgery and were sacrificed by CO_2_ asphyxiation at 24h post-CLP. The following criteria were used as humane endpoints; animals meeting any one of these criteria were considered moribund, counted as deceased in the enumeration of surviving animals, and sacrificed by CO_2_ asphyxiation. 1) Loss of 25% of body weight from baseline weight. 2) Major organ failure or medical conditions unresponsive to treatment such as severe respiratory distress, icterus, uremia, intractable diarrhea, or self-mutilation. 3) Surgical complications unresponsive to immediate intervention (bleeding, infection, wound dehiscence). 4) Clinical or behavioral signs unresponsive to appropriate intervention persisting for 24 hours including significant inactivity, labored breathing, sunken eyes, hunched posture, piloerection/matted fur, and abnormal vocalization when handled. No animals died within the 24 hour period until they were sacrificed in this study.

### Phenotypic flow cytometry

Mice were sacrificed and spleens were harvested 24-hours post CLP. Splenocytes were stained with anti-CD3-Alexa 700 (BD), anti-CD4-PB (BD), anti-CD8-PO and anti-CD44-PerCP (Biolegend). Cells were also surface stained with anti-CD25-FITC (Biolegend), anti-CD69-PE (Biolegend), anti-CD62L-PE Cy7 (BD), and anti-BTLA-PE, anti-2B4-APC, anti-PD-1-APC-Cy7, anti-LAG-3-FITC (all from eBioscience) for phenotypic analysis. An LSR II flow cytometer (BD Biosciences) was used to run all samples. Accucheck Counting Beads (Thermo Fisher Scientific) were added during staining to calculate the absolute number of T cells per spleen. Flow data were analyzed using FlowJo software (FlowJo, LLC) and SPADE (Cytobank.org) (see below).

### Intracellular cytokine staining

2x10^6^ splenocytes from each sample were plated in a 96-well plate. After centrifugation, cells were resuspended and incubated in culture medium (R10) consisting of RPMI 1640 containing 10% FBS (Mediatech, Herndon, VA), 2mM L-glutamine, 0.01 M HEPES buffer, 100mg/ml gentamicin (Mediatech), and 5×10^-5^M 2-mercaptoethanol (Sigma-Aldrich, St. Louis, MO). To test intracellular cytokine, cells were stimulated with 30 mg/ml PMA and 400 ng/ml ionomycin in the presence of GolgiStop (BD Pharmingen) for 4 hours at 37°C.

After incubation and stimulation, cells were surface-stained with anti-CD3-PB (BD), anti-CD4-PerCP (BD), anti-CD8-PO (Biolegend), anti-BTLA-PE, anti-2B4-APC, anti-PD-1-APC-Cy7 (all from eBioscience). Then cells were permeabilized using fixation and permeabilization solution (BD). We used anti-IL-2-FITC (BD), anti-TNF-PE-Cy7 (Biolegend) and anti-IFN-γ-Alexa 700 (BD) for intracellular cytokine staining. In some experiments, cells were stained intracellularly for Ki-67 using the Foxp3 staining kit (BD Pharmingen). Samples were analyzed on an LSRII flow cytometer (BD) and data were analyzed using FlowJo software (FlowJo, LLC) and SPADE (Cytobank.org) (see below).

### 2-NDBG assay

To assess the ability of CD4^+^ T cells from previously healthy vs. cancer septic animals to take up glucose, animals were sacrificed at 24h post-CLP and spleens were harvested. The cells were isolated and resuspended in a single cell solution in PBS. 2x10^6^ splenocytes were stained with anti-CD4 (BD Biosciences), anti-CD8 (Invitrogen) and anti-CD44 (eBioSciences) for 30 minutes at 4°C. Cells were washed twice in 250 μl of PBS and resuspended in 200 μl of 50 μM 2-NBDG (Thermofisher) and incubated at 37°C for 30 minutes. Cells were washed with PBS and analyzed on an LSRII flow cytometer (BD Biosciences). Data were analyzed using FlowJo 9 software (Treestar) and Prism 6 software (GraphPad Software Inc.).

### SPADE analysis

Traditional flow cytometry data was manually gated in FlowJo (FlowJo, LLC) on CD3^+^CD4^+^ lymphocytes and new FCS files were created containing only these gated events. The pre-gated data files were uploaded to Cytobank (Cytobank.org) for automated analysis by SPADE [39]. SPADE trees were generated using CD44, CD62L, CD69, CD25, and CXCR4 as the clustering channels for the activation panel; CD44, LAG-3, BTLA, 2B4, PD-1, and CD127 as the clustering channels for the exhaustion panel; and IL-2, IFN-γ, TNF, 2B4, PD-1, and BTLA as the clustering channels for the ICCS Panel. To simplify comparisons between previously healthy and cancer animals SPADE analysis utilized Fold Change Groups with the data files from previously healthy animals set as the baseline samples. The resulting SPADE trees were colored by the parameter “percent total ratio log” or log_10_ (percent of total sample/average percent of total baseline) to more easily visualize differences between cancer samples and the averaged baseline samples. The percent of total CD4^+^ cells in each node was compared between experimental groups and phenotypically similar nodes demonstrating significant differences between previously healthy and cancer animals were grouped into clusters for further analysis.

### Statistical analysis

All the data were expressed as mean ± SEM. The Student t test and Mann–Whitney U test were used to compare the continuous variables between two groups based on the Gaussian distribution. All statistical analyses were conducted using the GraphPad Prism 6.0 software (San Diego, CA). Two-tailed P-values < 0.05 were considered statistically significant.

## Results

### CD4^+^ T cells are increased in cancer septic mice as compared to previously healthy controls

**Fig 1:**
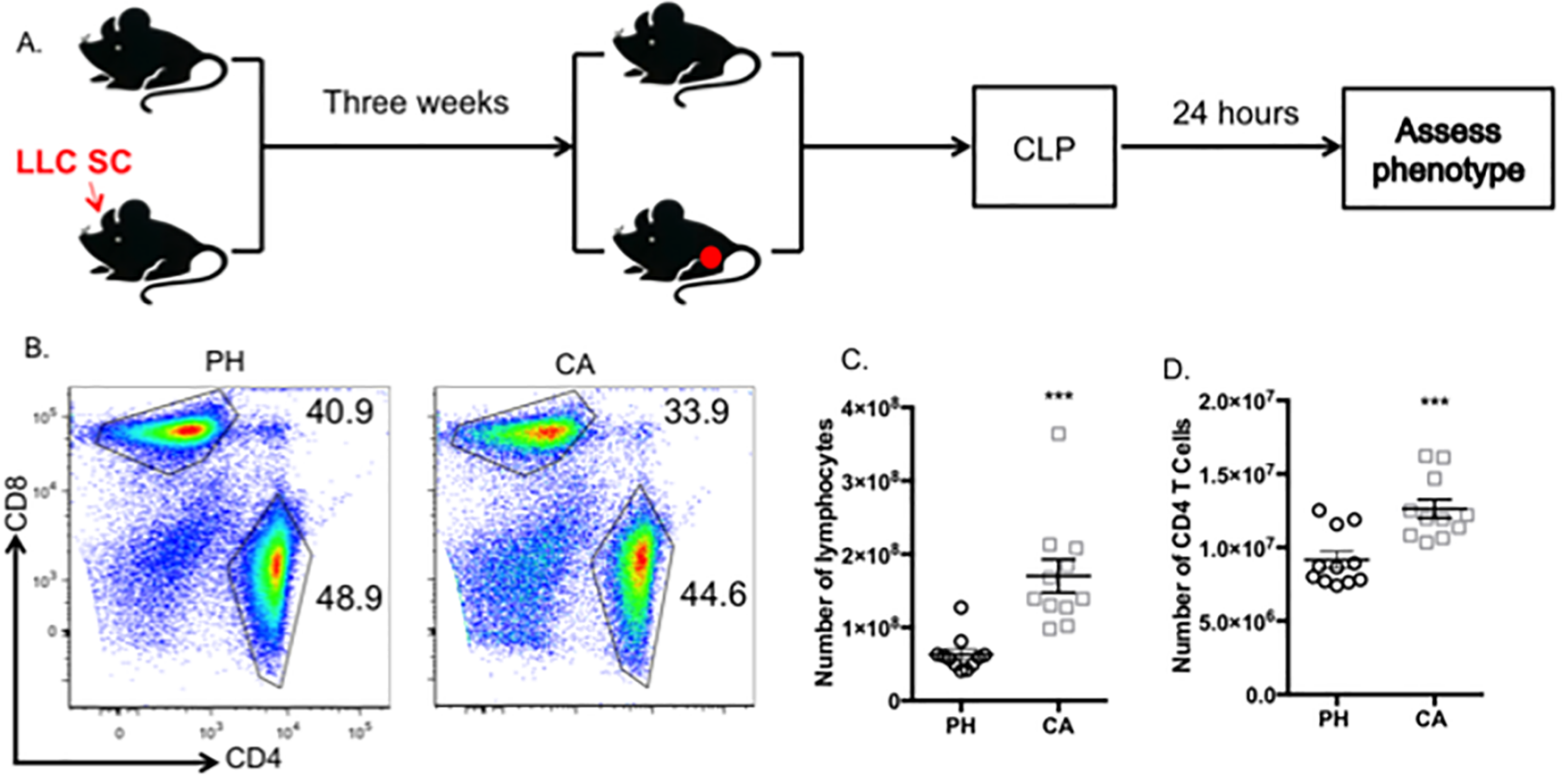
CD4^+^ T cells are increased in cancer septic mice as compared to previously healthy controls. Mice were injected with LLC tumor cells subcutaneously in the inner thigh as described in Materials and Methods. Tumors were allowed to grow for 3 weeks, at which time cancer mice and previously healthy age-matched controls were subjected to CLP (A). 24 h later, splenocytes were harvested and frequencies (B, C) and absolute numbers (D) of CD4+ T cells were assessed by flow cytometry. n = 11/group. ***p<0.001. PH = previously healthy septic. CA = cancer septic.

To determine the immunologic derangements associated with pre-existing malignancy during sepsis, we utilized an LLC1 mouse model of lung cancer [12, 14]. Animals were inoculated with LLC1 cancer cells as described in materials and methods and tumors were allowed to develop for three weeks. Cancer recipients or age- and gender-matched previously healthy controls were then subjected to CLP (Fig 1A). Twenty-four hours later, animals were sacrificed and spleens were analyzed by flow cytometry (Fig 1B). We observed a statistically significant increase in the absolute number of lymphocytes in the spleens of cancer septic animals as compared to previously healthy septic controls (Fig 1C). While CD8^+^ T cells were modestly increased, we identified a significant increase in the absolute number of CD4^+^ T cells in cancer septic hosts as compared to previously healthy controls (Fig 1D). Owing to this observation and the known critical role of CD4^+^ T cells in sepsis immune dysfunction [40], we chose to further interrogate the phenotypes and function of CD4^+^ T cells in this system.

### SPADE analysis identifies three primary clusters that distinguish CD4^+^ T cells from cancer septic mice as compared to previously healthy controls

**Fig 2:**
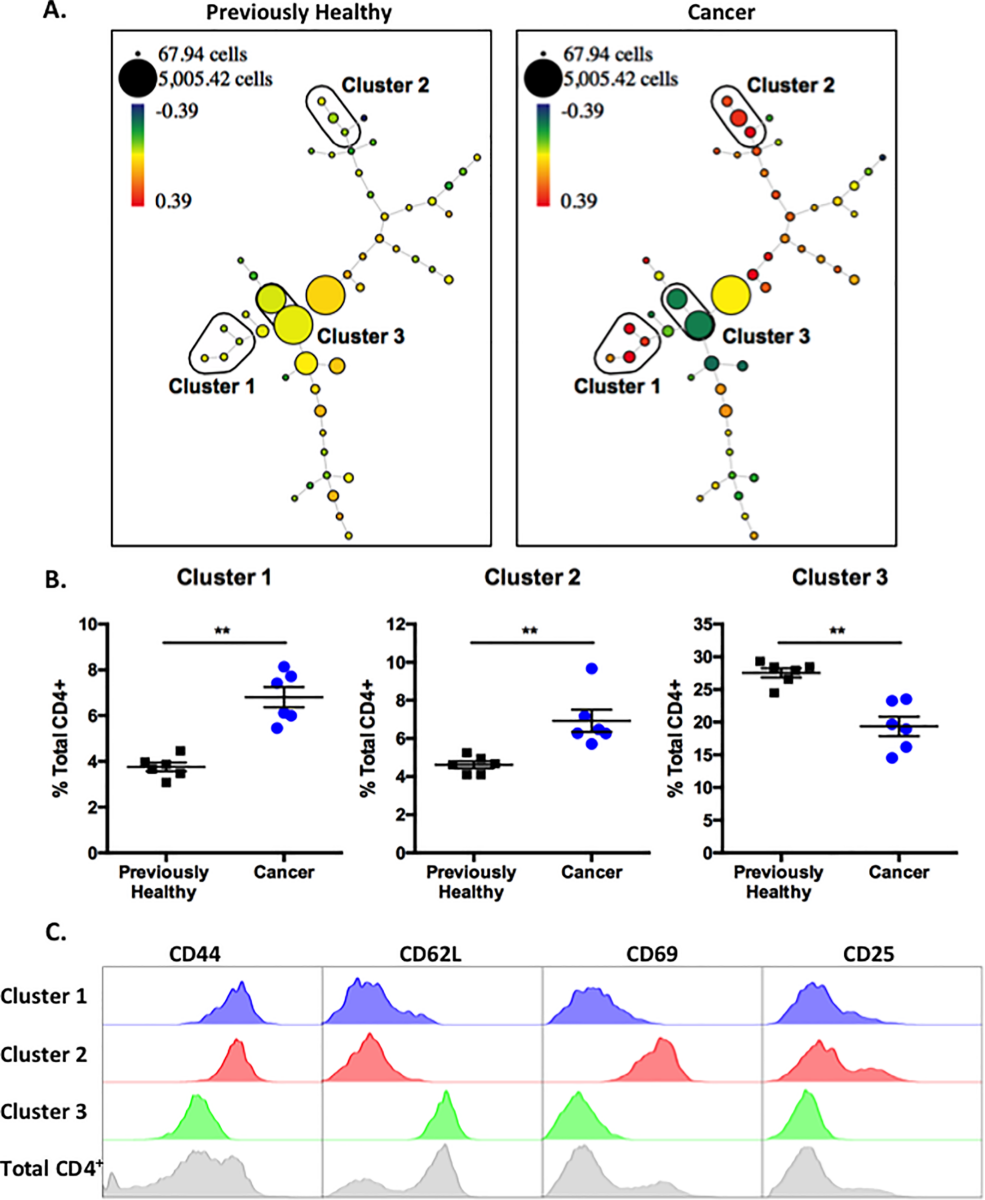
SPADE analysis identifies three primary clusters that distinguish CD4^+^ T cells from cancer septic mice as compared to previously healthy controls. Traditional flow cytometry data generated from cells in Fig 1 were manually gated in FlowJo on CD3^+^CD4^+^ lymphocytes and new FCS files were created containing only these gated events. The pre-gated data files were uploaded to Cytobank and SPADE trees were generated using CD44, CD62L, CD69, CD25, and CXCR4 as the clustering channels. A, Representative SPADE trees are shown. Fold Change Groups was used to make comparisons with the data files from previously healthy animals set as the baseline samples. SPADE trees depicted are colored by the parameter “percent total ratio log” or log_10_ (percent of total sample/average percent of total baseline). The percent of total CD4^+^ cells in each node was compared between previously healthy and cancer septic animals, and phenotypically similar nodes demonstrating significant differences between previously healthy and cancer animals were grouped into clusters for further analysis. B. Frequencies of total CD4^+^ T cells falling into each Cluster within each experimental group are depicted. n = 6/group, representative of two independent experiments with a total of n = 11/group. C, The expression patterns of CD44, CD62L, CD69, and CD25 within each Cluster are depicted.

In order to conduct a non-biased, unsupervised analysis of phenotypic differences between the cancer and non-cancer septic groups, we used SPADE (Spanning-tree Progression Analysis of Density-normalized Events) analysis to identify populations of cells most different between the two groups. SPADE is a method to identify populations in an unsupervised manner in multidimensional flow cytometry data files, to cluster populations into nodes, and project them into a tree (Fig 2A)[39]. Each node contains cells with the same phenotype across all parameters analyzed, and the size of the node indicates the number of cells contained within that population, to facilitate comparison between animals. To compare CD4^+^ T cell populations between the cancer and previously healthy septic groups, flow cytometry data was manually gated in FlowJo on CD3^+^CD4^+^ lymphocytes and new FCS data files were created containing only these gated events. The pre-gated data files were uploaded to Cytobank (Cytobank.org) for automated analysis by SPADE [39]. SPADE trees were generated using CD44, CD62L, CD69, CD25, and CXCR4 as the clustering channels. To compare between previously healthy and cancer animals, SPADE analysis utilized Fold Change Groups with the data files from previously healthy animals set as the baseline samples. To display the differences between the groups, SPADE trees are colored by the parameter “percent total ratio log” or log_10_ (percent of total sample/average percent of total baseline) (Fig 2A).

After performing this analysis, we compared the percent of total CD4^+^ cells in each node between previously healthy and cancer animals and identified 3 clusters of nodes that were significantly different between CD4^+^ T cells isolated from previously healthy septic animals (representative animal shown in Fig 2A, left panel) as compared to cancer septic animals (representative animals shown in Fig 2A, right panel). Clusters 1 and 2 were increased in cancer septic mice relative to non-cancer septic controls, and Cluster 3 was decreased in cancer septic mice relative to non-cancer septic controls. Differences in cell populations residing in these three clusters were reproducible among animals, as evidenced by summary data shown in Fig 2B. Finally, we queried the phenotypes of the cell populations within each SPADE cluster. Using the phenotypes of total CD4^+^ T cells as a reference (Fig 2C, gray histograms, bottom row), we found that Cluster 1 contained cells that were CD44^hi^, CD62L^lo^, CD69^lo^, and CD25^lo^, a phenotype suggestive of resting memory T cells. Cluster 2 contained cells that were CD44^hi^, CD62L^lo^, CD69^hi^, and CD25^hi^, a phenotype that was suggestive of recently activated effectors. Finally, Cluster 3 contained cells that were CD44^lo^, CD62L^hi^, CD69^lo^, and CD25^lo^, a phenotype suggestive of resting naïve CD4^+^ T cells. In sum, SPADE analysis demonstrates that relative to non-cancer controls, cancer mice contained more resting memory CD4^+^ T cells, more activated CD4^+^ effectors, and fewer naïve CD4^+^ T cells during sepsis.

**Fig 3:**
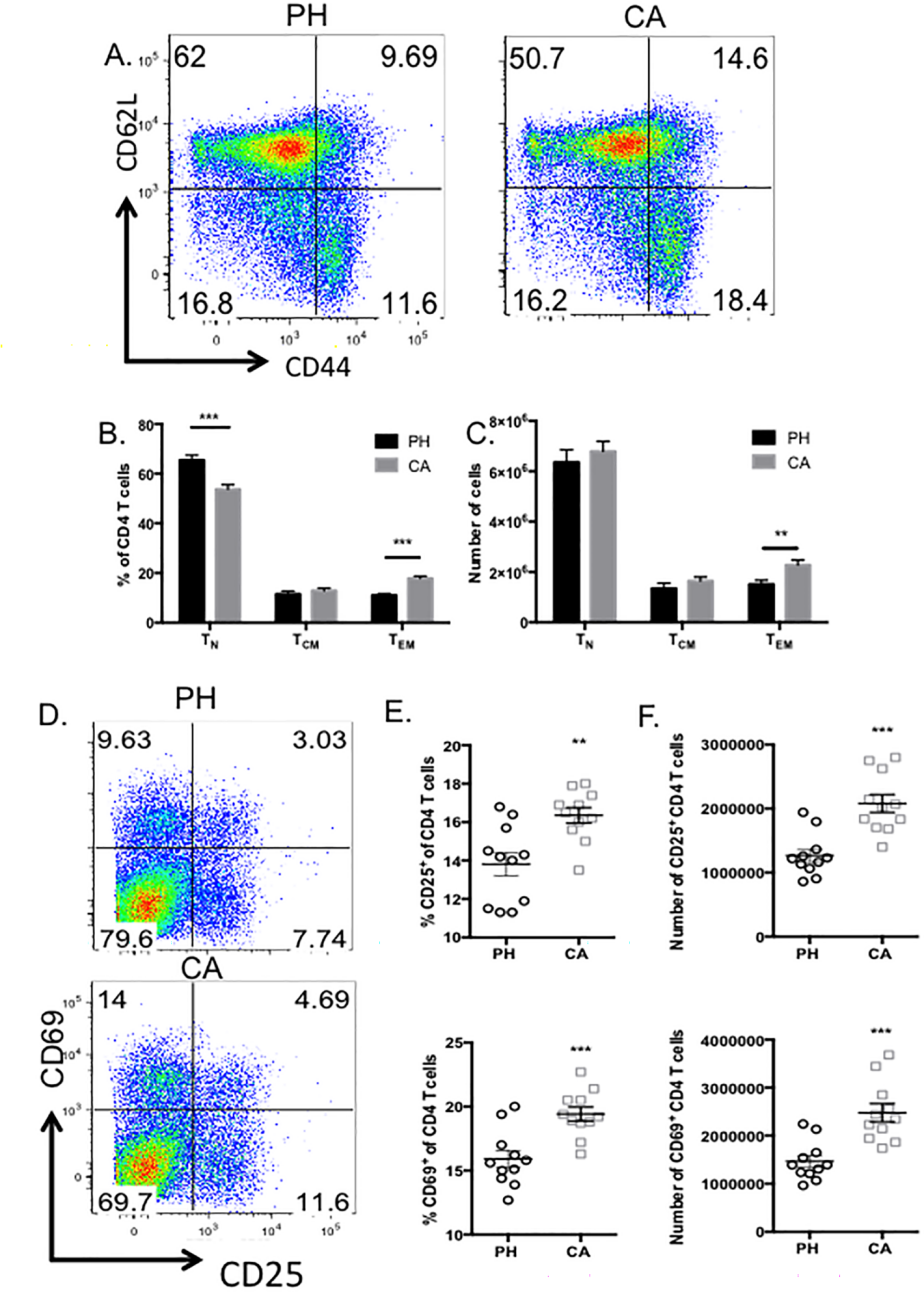
Increased frequencies and absolute numbers of effector memory CD4^+^ T cells in cancer septic animals relative to previously healthy septic controls. CD4^+^ T cells isolated from splenocytes of previously healthy or cancer septic animals were analyzed by flow cytometry. A, Representative flow plots of CD44 and CD62L expression. Frequencies (B) and absolute numbers (C) of naïve, central memory, and effector memory T cells from previously healthy vs. cancer septic animals. D, Representative flow plots of CD69 and CD25 expression. Frequencies (E) and absolute numbers (F) of CD25^+^ and CD69^+^ CD4^+^ T cells from previously healthy vs. cancer septic animals. n = 11/group. ***p<0.001.

We next sought to verify these SPADE results using traditional FlowJo analysis. CD4^+^ T cells were gated by CD62L and CD44 expression to identify naïve (CD44^lo^ CD62L^hi^), central memory (T_CM_, CD44^hi^ CD62L^hi^), effector memory (T_EM_, CD44^hi^ CD62L^lo^) (Fig 3A). Results indicated a statistically significant decrease in the frequency of naïve CD4^+^ T cells (Fig 3B) and a significant increase the frequency of T_EM_ (Fig 3B) that was also reflected in the increase in absolute numbers (Fig 3C). Moreover, we also identified an increase in both the frequency and absolute number of CD25^+^ cells and CD69^+^ cells (Fig 3D–3F), markers which are likely indicative of recent activation.

### CD4^+^ T cell populations in cancer septic hosts are characterized by the increased prevalence of two distinct populations of cells

**Fig 4:**
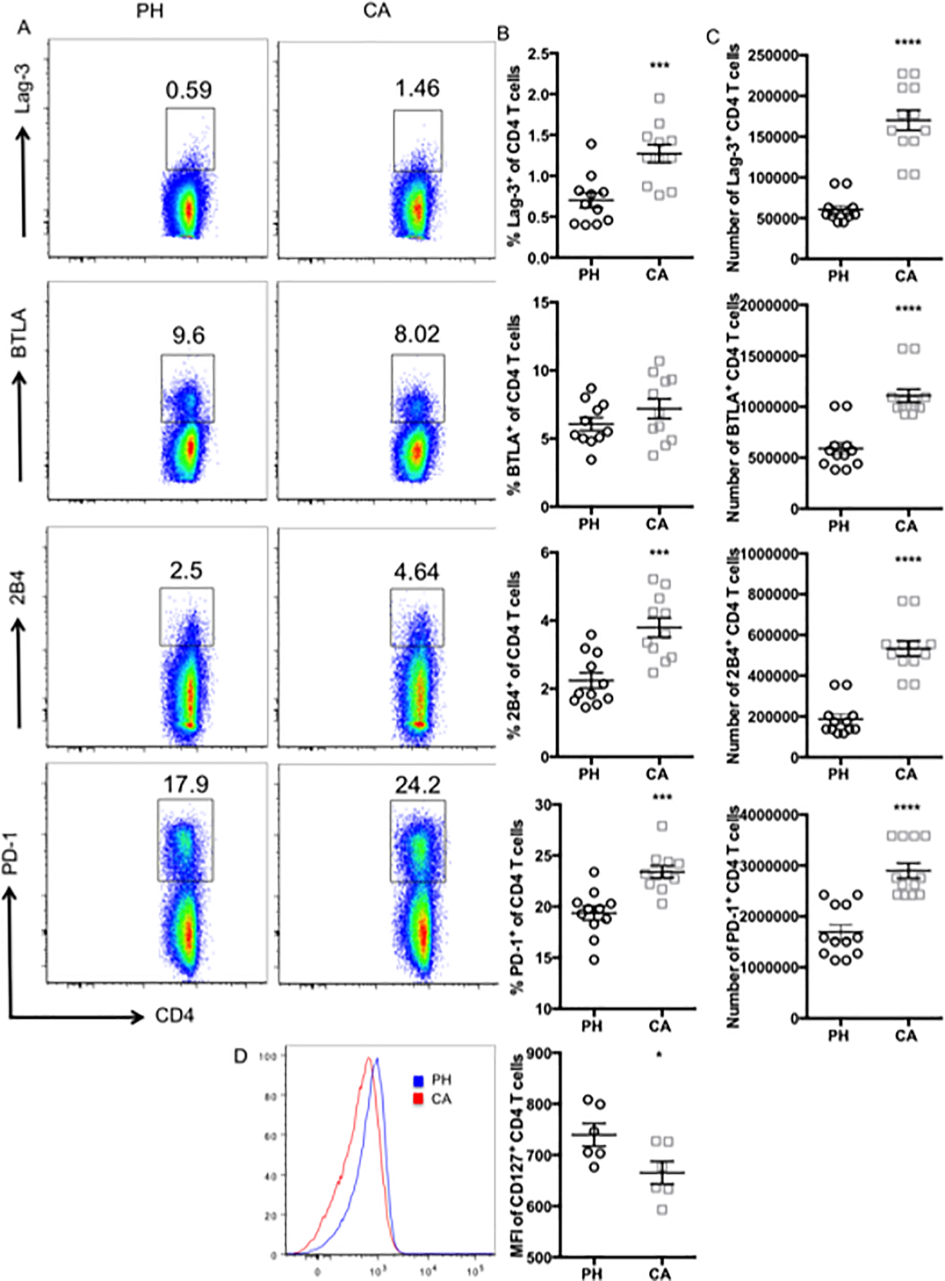
CD4^+^ T cell populations in cancer septic animals exhibit increased coinhibitory receptor expression relative to previously healthy septic controls. CD4^+^ T cells isolated from splenocytes of previously healthy or cancer septic animals were analyzed for coinhibitory receptor expression by flow cytometry. A, Representative flow plots of LAG-3, BTLA, 2B4, and PD-1 expression. Frequencies (B) and absolute numbers (C) of LAG-3^+^, BTLA^+^, 2B4^+^, and PD-1^+^ CD4^+^ T cells from previously healthy vs. cancer septic animals. D, Representative flow plots of CD127 expression. MFI of CD127 expression on CD4^+^ T cells from previously healthy vs. cancer septic animals. n = 11/group. ***p<0.001, *p<0.05.

Given the propensity of tumors to increase T cell expression of exhaustion markers within the tumor microenvironment, we next interrogated the systemic expression of common exhaustion markers (LAG-3, BTLA, 2B4, PD-1, and TIM-3) on splenic CD4^+^ T cells at 24 h post-sepsis induction in cancer vs. non-cancer hosts. CD127 (IL-7Ra) was also included because exhausted T cells have been described to exhibit reduced expression of this cytokine receptor [41, 42]. Traditional flow cytometric analysis in FlowJo revealed that LAG-3, 2B4, and PD-1 were all significantly upregulated on CD4^+^ T cell populations in cancer septic mice relative to previously healthy control mice (Fig 4A and 4B), resulting in significant increases in the absolute numbers of CD4^+^ T cells expressing these phenotypic markers of exhaustion (Fig 4C). In contrast, expression of CD127 was downregulated in CD4^+^ T cell populations in cancer septic mice relative to previously healthy control mice (Fig 4D).

**Fig 5:**
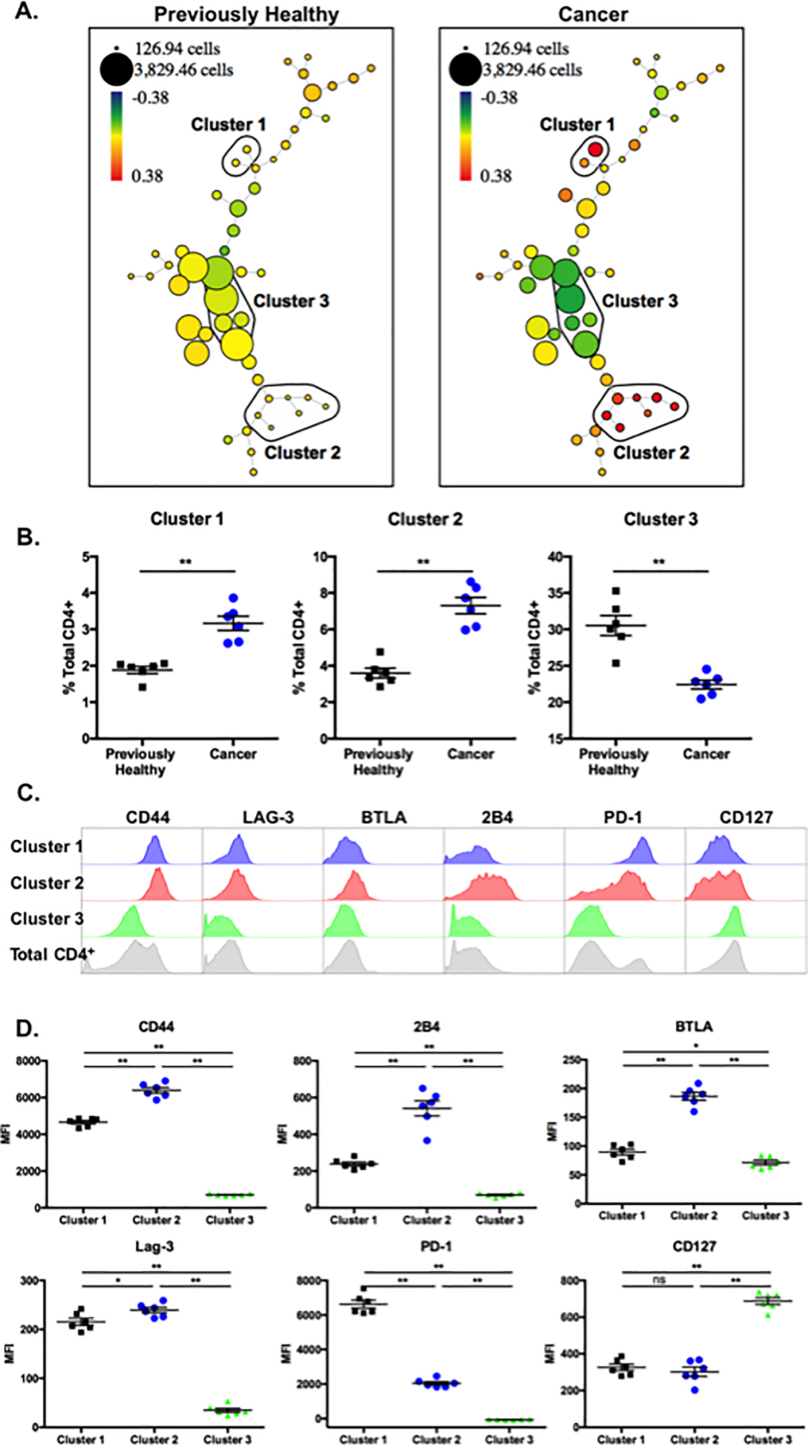
CD4^+^ T cell populations in cancer septic hosts are characterized by the increased prevalence of two distinct populations of cells. Traditional flow cytometry data generated from cells in Fig 1 were manually gated in FlowJo on CD3^+^CD4^+^ lymphocytes and new FCS files were created containing only these gated events. The pre-gated data files were uploaded to Cytobank and SPADE trees were generated using CD44, LAG-3, BTLA, 2B4, PD-1, and CD127 as the clustering channels. A, Representative SPADE trees are shown. Fold Change Groups was used to make comparisons with the data files from previously healthy animals set as the baseline samples. SPADE trees depicted are colored by the parameter “percent total ratio log” or log_10_ (percent of total sample/average percent of total baseline). The percent of total CD4^+^ cells in each node was compared between previously healthy and cancer septic animals, and phenotypically similar nodes demonstrating significant differences between previously healthy and cancer animals were grouped into clusters for further analysis. B. Frequencies of total CD4^+^ T cells falling into each Cluster within each experimental group are depicted. n = 6/group, representative of two independent experiments with a total of n = 11/group. C, The expression patterns of CD44, LAG-3, BTLA, 2B4, PD-1, and CD127 within each Cluster are depicted. D, Summary data of MFI of CD44, LAG-3, BTLA, 2B4, PD-1, and CD127 on the cells in each Cluster for n = 6 mice/group are shown. **p<0.01.

To attempt to identify more granular differences in CD4^+^ T cell populations expressing exhaustion markers in cancer septic hosts, SPADE analysis was performed on data generated from flow cytometric analysis of the exhaustion markers listed above. These analyses identified 3 clusters of nodes that are significantly different between previously healthy controls (Fig 5A, left panel) and cancer septic animals (Fig 5A, right panel). Clusters 1 and 2 were significantly increased in cancer septic hosts, and Cluster 3 was significantly decreased in cancer septic hosts (Fig 5B). Further analysis shown in Fig 5C revealed the identity of cells within each of the three clusters, relative to the total CD4^+^ T cell population (grey histograms): Cluster 1 was comprised of cells that were CD44^hi^ LAG-3^hi^ BTLA^int^ 2B4^int^ PD-1^hi^ CD127^lo^. Cluster 2 was comprised of cells that were CD44^hi^ LAG-3^hi^ BTLA^hi^ 2B4^hi^ PD-1^int^ CD127^int^. In contrast, Cluster 3 was comprised of cells that were CD44^lo^ LAG-3^lo^ BTLA^lo^ 2B4^lo^ PD-1^lo^ CD127^hi^. Thus, SPADE analysis of phenotypic markers of exhaustion revealed that mice with pre-existing malignancy exhibit a significant increase in the expression of exhaustion markers during sepsis relative to previously healthy counterparts during sepsis. Importantly, this analysis illuminated two distinct clusters of CD4^+^ T cells bearing phenotypic markers of exhaustion. As shown in Fig 5D, cells in Cluster 1 express significantly more PD-1 than do those in Cluster 2, and cells in Cluster 2 express significantly more 2B4, BTLA, LAG-3 and CD44 than those in Cluster 1. Thus, SPADE analysis facilitates a more granular view into the phenotypic derangements on CD4^+^ T cells observed in mice with pre-existing malignancy following sepsis. These analyses therefore highlight the fact that although many coinhibitory receptors were upregulated when examined in bulk, the PD-1^hi^ population is distinct from the 2B4^hi^ BTLA^hi^ LAG-3^hi^ population in cancer septic animals.

### Differential functionality of distinct CD4^+^ T cell populations in cancer septic animals relative to previously healthy septic controls

**Fig 6:**
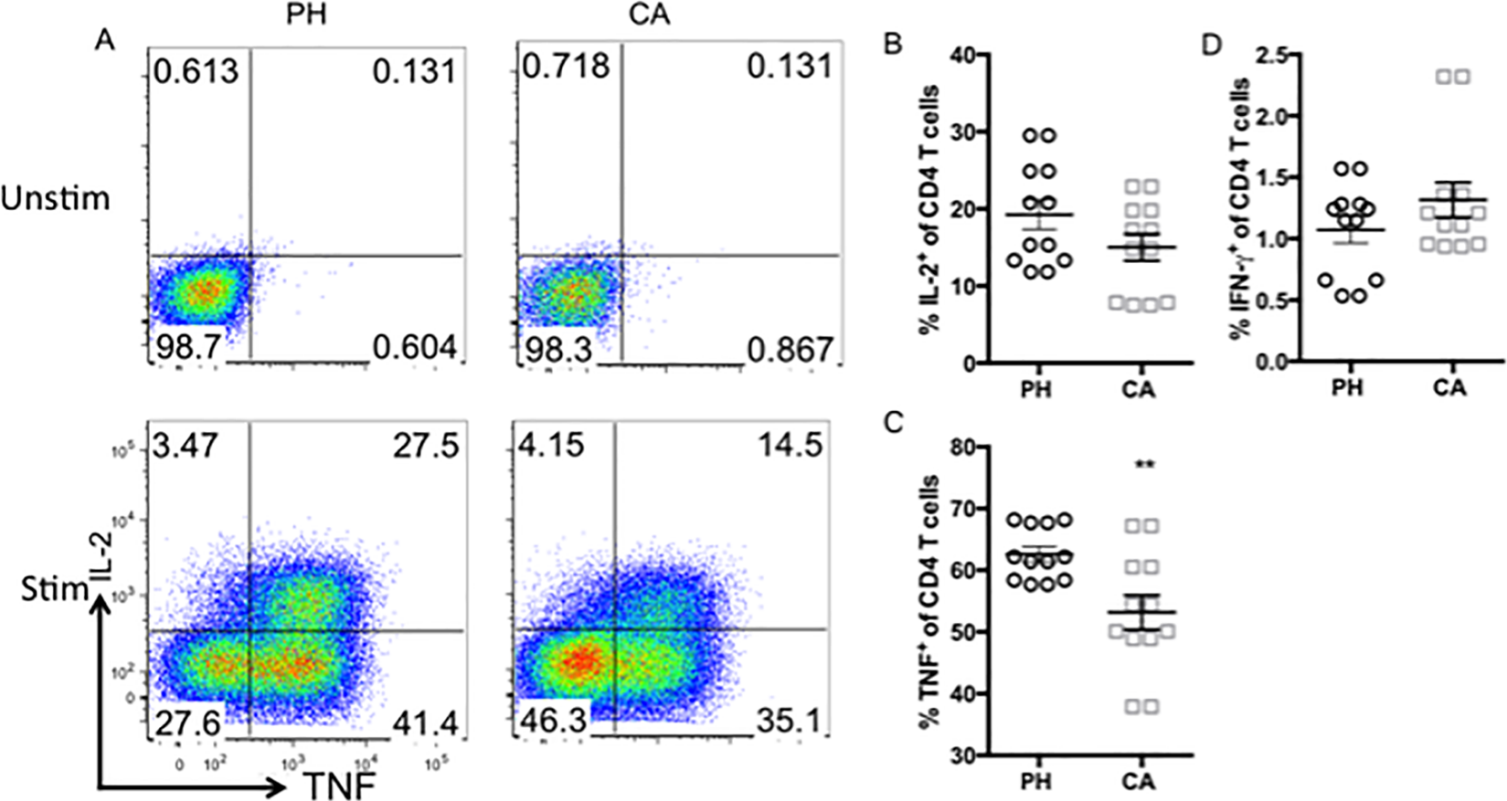
CD4^+^ T cell populations in cancer septic animals exhibit increased TNF secretion relative to previously healthy septic controls. CD4^+^ T cells isolated from splenocytes of previously healthy or cancer septic animals were restimulated ex vivo with PMA/ionomycin as described in materials and methods and stained intracellularly for IL-2, TNF, and IFN-γ. A, Representative flow plots of IL-2 and TNF expression in unstimulated (top panels) and stimulated (bottom panels) cells isolated from previously healthy (left panels) vs. cancer septic (right panels) mice. B-D, Summary data of frequencies of IL-2^+^ (B), TNF^+^ (C), and IFN-γ^+^ (D) CD4^+^ T cells from previously healthy (PH) vs. cancer (CA) septic animals. n = 11/group. **p<0.01.

We next sought to determine the functionality of CD4^+^ T cell populations in cancer vs. non-cancer hosts during sepsis. Splenocytes from the two groups were isolated at 24 h post CLP, and cells were restimulated ex vivo for 4 hours as described in materials and methods. Frequencies of IFN- γ, TNF, and IL-2 producers were assessed using traditional FlowJo analysis (Fig 6A). Results indicated that there was no difference in the frequency of IFN-γ or IL-2 producers between cancer and non-cancer hosts during sepsis (Fig 6B and 6D). In contrast, we identified a significant decrease in the frequency of TNF producers in cancer septic mice relative to previously healthy septic controls (Fig 6A and 6C).

**Fig 7:**
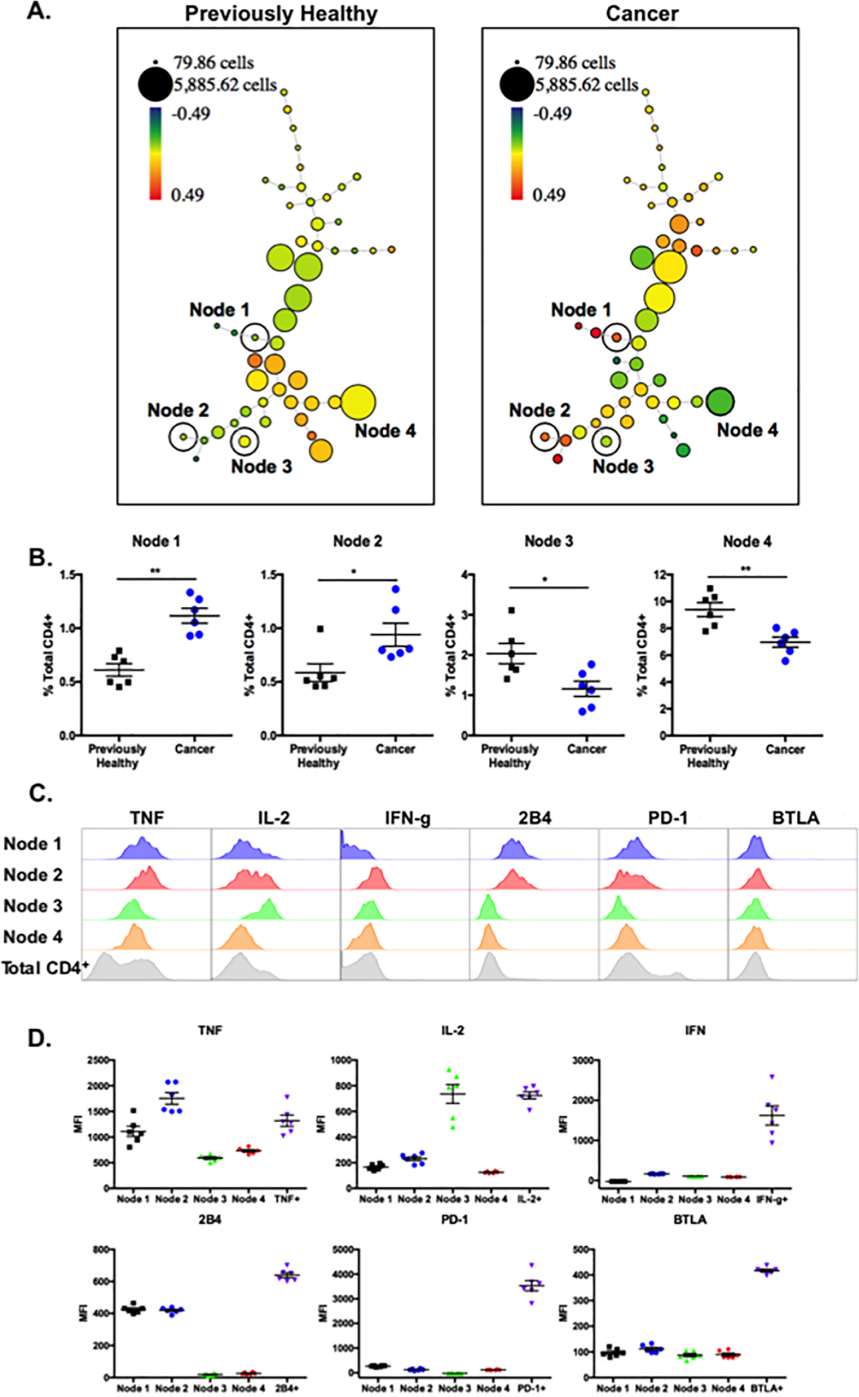
Differential functionality of distinct CD4^+^ T cell populations in cancer septic animals relative to previously healthy septic controls. Traditional flow cytometry data generated from cells in Fig 6 were manually gated in FlowJo on CD3^+^CD4^+^ lymphocytes and new FCS files were created containing only these gated events. The pre-gated data files were uploaded to Cytobank and SPADE trees were generated using TNF, IL-2, IFN-γ, BTLA, 2B4, and PD-1 as the clustering channels. A, Representative SPADE trees are shown. Fold Change Groups was used to make comparisons with the data files from previously healthy animals set as the baseline samples. SPADE trees depicted are colored by the parameter “percent total ratio log” or log_10_ (percent of total sample/average percent of total baseline). The percent of total CD4^+^ cells in each node was compared between previously healthy and cancer septic animals, and phenotypically similar nodes demonstrating significant differences between previously healthy and cancer animals were analyzed further. B. Frequencies of total CD4^+^ T cells falling into each Node within each experimental group are depicted. n = 6/group, representative of two independent experiments with a total of n = 11/group. C, The expression patterns of TNF, IL-2, IFN-γ, BTLA, 2B4, and PD-1 within each Node are depicted. D, Summary data of MFI of TNF, IL-2, IFN-γ, BTLA, 2B4, and PD-1 on the cells in each Node for n = 6 mice/group are shown. The MFIs of cells positive for each parameter (derived from other nodes) are also depicted as positive controls.

We next analyzed the same data using SPADE. Comparison of previously healthy septic vs. cancer septic hosts across parameters that included IFN-γ, TNF, IL-2, PD-1, 2B4, and BTLA generated the SPADE trees shown in Fig 7A (LAG-3 and CD127 could not be included due to panel limitations). Results revealed 4 cytokine-producing nodes that were differentially represented in previously healthy septic animals (left panel) as compared to cancer septic animals (right panel). Other nodes showing differences between the previously healthy and cancer groups were not found to be producing any cytokine and merely recapitulated the differences seen in 2B4 and PD-1 as described in Fig 5. Nodes 1 and 2 were significantly increased in cancer septic hosts, and Nodes 3 and 4 were significantly decreased in cancer septic hosts (Fig 7B). Further analysis shown in Fig 7C revealed the identity of cells within each of the three clusters, relative to the total CD4^+^ T cell population (grey histograms): Node 1 was comprised of cells that were TNF^int^ IL-2^lo^ IFN- γ^lo^ 2B4^int^ PD-1^lo^BTLA^lo^, indicative of cells that are both phenotypically and functionally exhausted. Node 2 was comprised of cells that were TNF^hi^ IL-2^int^ IFN- γ^lo^ 2B4^int^ PD-1^lo^ BTLA^lo^, indicative of cells that expressed some phenotypic markers of activation/exhaustion but retained cytokine-secreting capacity. In contrast, Node 3 was comprised of cells that were TNF^lo^ IL-2^hi^ IFN- γ^lo^ 2B4^lo^ PD-1^lo^ BTLA^lo^, suggestive of non-exhausted cells that retain proliferative capacity as evidenced by their high expression of IL-2. Finally, Node 4 was comprised of cells that were TNF^lo^ IL-2^lo^ IFN- γ^lo^ 2B4^lo^ PD-1^lo^ BTLA^lo^, suggestive of naïve or weakly activated T cells. Thus, SPADE analysis also facilitates a more granular view into the functional derangements in CD4^+^ T cells observed in mice with pre-existing malignancy following sepsis. As summarized in Fig 7D, we conclude that cancer septic animals exhibit an increase in a subset of 2B4^+^ PD-1^lo^ TNF-secreting CD4^+^ T cells (i.e. Nodes 1 and 2) relative to previously healthy septic controls. On the other hand, cancer septic animals exhibit a decrease in a subset of PD-1^lo^ 2B4^lo^ IL-2-secreting CD4^+^ T cells (i.e. Nodes 3 and 4) relative to previously healthy septic controls.

### Cancer sepsis impacts CD4^+^ T cell proliferation but not glucose uptake

**Fig 8:**
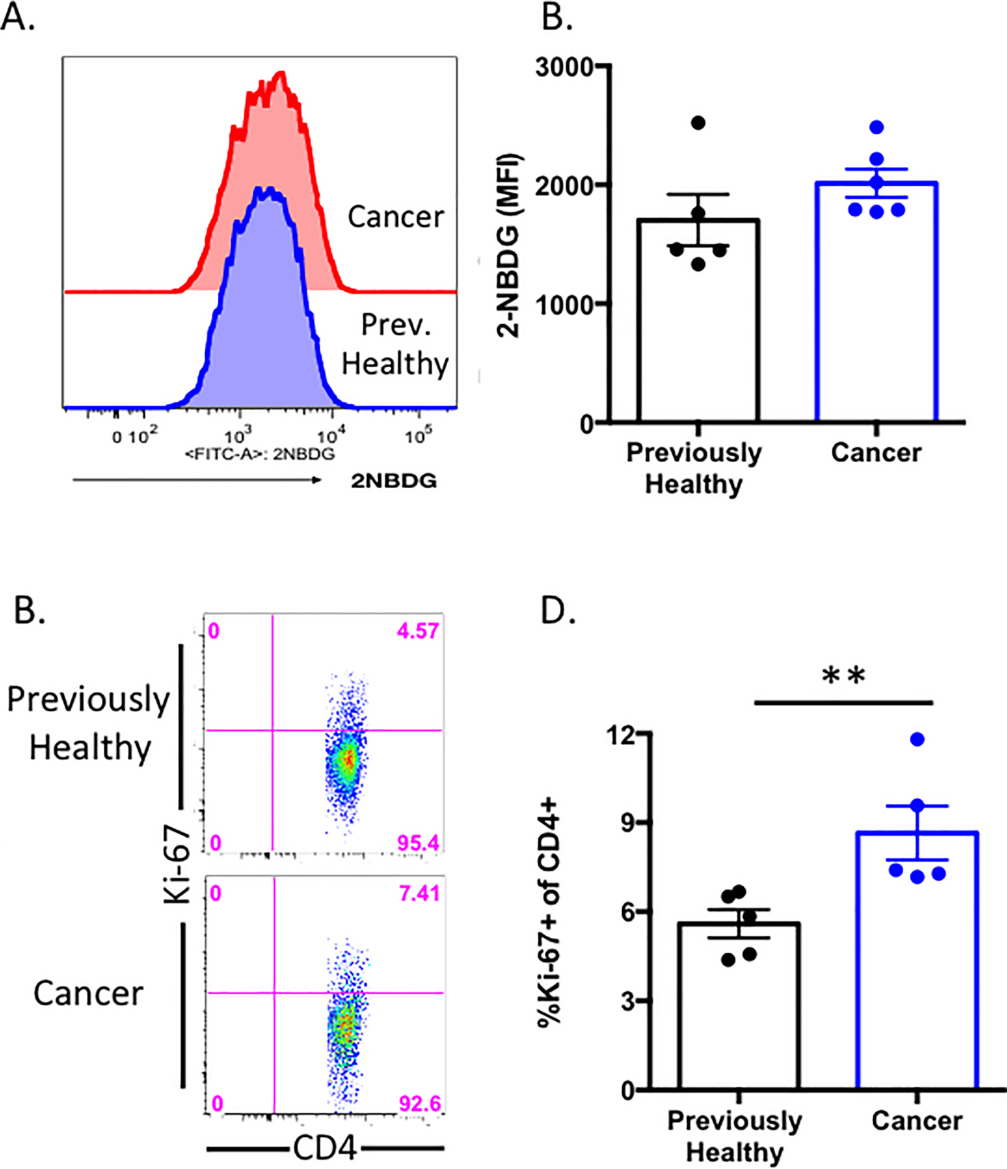
Cancer sepsis impacts CD4^+^ T cell proliferation but not glucose uptake. CD4^+^ T cells isolated from splenocytes of previously healthy or cancer septic animals were A) incubated for 30 min ex vivo with fluorescently labeled 2-NBDG as described in Materials and Methods or B) stained intracellularly for Ki-67 as described in Materials and Methods. A, Representative flow plots of 2-NBDG uptake in CD4^+^ T cells cells isolated from previously healthy (blue histograms) vs. cancer septic (red histograms) mice. B, Summary data of frequencies of 2-NBDG^+^ CD4^+^ T cells. (C) Representative flow plots and D) summary data of Ki-67 staining in CD4^+^ T cells populations isolated from previously healthy vs. cancer septic mice. Summary data tabulated from n = 5/group. **p<0.01.

Our data identified an increase in activated CD4^+^ T cells and a subset that exhibited increased TNF production. We next queried the impact of cancer sepsis on CD4^+^ T cell proliferation and metabolic function. Using the same experimental setup, splenocytes obtained from previously healthy septic vs. cancer septic mice at 24h post CLP were stained directly ex vivo with Ki-67 to identify recently proliferated cells. Additional aliquots of cells were cultured for 30 min with the fluorescent glucose analog 2-NBDG as described in Materials and Methods in order to determine the degree of glucose uptake in CD4^+^ T cell populations isolated from the two groups. Interestingly, our data show that while there was no difference in uptake of 2-NDBG between previously healthy septic and cancer septic animals (Fig 8A), we identified a statistically significant increase in the frequency of Ki-67^+^ cells within the CD4^+^ T cell compartment of cancer septic animals as compared to previously healthy septic animals (Fig 8B). These data highlight a functional difference in the CD4^+^ T cells in cancer septic vs. previously healthy septic hosts, and suggest at least in some subsets, the upregulation of molecules such as PD-1, 2B4, etc. may be markers of increased immune activation in cancer septic animals. This possibility is further supported by the finding of increased TNF secretion by CD4^+^ T cells in cancer septic as compared to previously healthy septic hosts (Fig 7).

## Discussion

Our study used two methods of analysis of flow cytometry data (FlowJo and SPADE) to interrogate the phenotypic and functional differences in CD4^+^ T cells between mice undergoing a septic insult that are previously healthy vs. those that have pre-existing malignancy. In general, there was good agreement between the two platforms in terms of identifying differences between the two groups. For example, both analysis methods identified the increased frequency of T_EM_ phenotype CD44^hi^ CD62L^lo^ in cancer septic animals relative to previously healthy controls, as well as increased expression of markers of acute activation CD69 and CD25. Moreover, we identified an increased frequency of Ki-67^+^ divided CD4^+^ T cells in cancer septic hosts relative to previously healthy septic hosts. Thus, we conclude that the CD4^+^ T cell compartment in cancer septic hosts is one of increased activation and differentiation.

The fact that cancer septic hosts, which exhibit increased mortality during sepsis, have increased frequencies of memory T cells is of interest when considered in light of our recently published results in which we found that attrition of memory T cells was deleterious during sepsis [43]. Specifically, we found that decreased numbers of “resting” memory T cells (those that had not upregulated CD25 and CD69) were associated with increased serum levels of IL-6 at 6 h post-CLP, which has been shown to predict mortality [44, 45]. Thus, increased frequencies of resting memory T cells in cancer septic hosts may confer a greater proportion of the CD4^+^ T cell compartment that is most susceptible to dysregulation in the context of sepsis.

Furthermore, both analysis strategies identified significant upregulation of multiple phenotypic markers of exhaustion in the setting of cancer and sepsis relative to previously healthy controls. Indeed, the expression of multiple distinct coinhibitory receptors has been shown to function in aggregate to contribute to increased functional exhaustion in antigen-specific CD8^+^ T cells [46, 47]. However, SPADE analysis revealed two distinct clusters of cells, both upregulated in the setting of cancer sepsis and both expressing phenotypic markers of exhaustion: one that expresses more PD-1 and one that expresses more 2B4, BTLA, and LAG-3. Thus, these results suggest that there may be multiple distinct phenotypes that are generated in cancer septic animals. It is not known if either or both of these subsets plays a causal role in the increased mortality and immune dysregulation observed in the setting of cancer and sepsis. However, identifying specific and differential phenotypes may facilitate targeting one or the other to determine their functional role during sepsis, and to potentially facilitate more targeted immunotherapy during sepsis. Moreover, our analyses identifying subsets of cells expressing multiple coinhibitory receptors raise the possibility that T cells in cancer mice may be subject to the control of more than one coinhibitory molecule, and that restoring their function may require the blockade of more than one coinhibitory pathway.

One limitation of this phenotypic analysis is that we did not exclude Foxp3-expressing cells from the analysis. Because Foxp3^+^ Treg constitute approximately 10% of the CD4^+^ T cell compartment in these animals, it is possible that some of the differences in populations we observed are comprised in whole or in part of Foxp3-expressing cells. Given their known roles in the pathogenesis of both cancer and sepsis individually [15, 48–52], interrogating the numbers, phenotypes, and functional changes in Foxp3^+^ Treg populations in the setting of cancer and sepsis remains an important area of future research.

Similar to the analysis of phenotypic markers, analysis of cytokine secreting capacity revealed that the condition of cancer and sepsis resulted in an overall decrease in TNF producing cells. However the observed increase in highly productive TNF-secreting CD4^+^ T cells, raises the possibility that these TNF-secreting populations of T cells could be pathophysiologic for septic animals with pre-existing malignancy. Interestingly, these TNF-secreting subsets did not express appreciable PD-1. This is of interest because PD-1 blockers are currently in clinical trials for sepsis, however the impact of PD-1 antagonism in the setting of cancer and sepsis is unknown. If PD-1 is not expressed on the specific T cell subsets underlying the immune dysregulation and mortality present in the setting of cancer and sepsis, then it is possible that anti-PD-1 may be less functional in this context. In contrast, our data suggest that TNF antagonism, which was tested in clinical trials in sepsis and failed [53, 54], could be revisited in the context of sepsis with pre-existing malignancy. Thus, while it is true that the overall immunophenotypic landscape of sepsis in the setting of cancer is characterized by increased expression of phenotypic markers of exhaustion, the CD4^+^ T cell compartment in these animals is heterogenous, with increased populations of both exhausted CD4^+^ T cells expressing PD-1 and activated CD4^+^ T cells exhibiting increased TNF secretion, both relative to non-cancer septic controls. Further investigation is required to determine which of these subsets are causally responsible for the increased mortality observed during sepsis.
